# Long-term endurance exercise influences exercise capacity in mice through modulation of the intestinal microbiota by fecal microbiota transplantation

**DOI:** 10.3389/fmicb.2026.1830033

**Published:** 2026-06-29

**Authors:** Wenqian Yang, Yuqian Liu, Haitao Wang, Guang Yang

**Affiliations:** 1 Institute of Exercise and Health, Lingnan Normal University, Zhanjiang, Guangdong, China; 2 School of Sports Science, Lingnan Normal University, Zhanjiang, Guangdong, China

**Keywords:** exercise capacity, fecal microbiota transplantation, intestinal microbiota, long-term endurance exercise, metabolic function

## Abstract

**Background:**

Exercise alters intestinal microbiota composition; however, it remains unclear whether these alterations induced by long-term endurance exercise can be transferred by fecal microbiota transplantation (FMT) to improve recipient exercise capacity.

**Methods:**

Mice were divided into control (Group C), exercise (Group E), PBS transplantation (Group PT), and FMT (Group MT) groups. Group E underwent 14 weeks of treadmill training, and Groups PT and MT were transplanted with PBS or fecal microbiota from Group E, respectively. Subsequently, exhaustive exercise tests and 16S rRNA sequencing were performed, and blood glucose levels, glycogen reserves, and antioxidant indices were assessed.

**Results:**

Compared with Group C, Group E had significantly higher hepatic/muscle glycogen levels and superoxide dismutase activity, and lower liver malondialdehyde content. After transplantation, Group MT showed significantly higher *Firmicutes* abundance, alpha diversity (Shannon and Simpson indices), and enrichment of eight beneficial genera (e.g., *Bifidobacterium*, *Dorea*) than Group PT, along with lower abundance of two harmful/conditional genera (e.g., *Sutterella*, *Parabacteroides*). Forty Kyoto Encyclopedia of Genes and Genomes metabolic pathways (e.g., starch and sucrose metabolism, flavone and flavonol biosynthesis) differed significantly between the PT and MT groups. The exhaustive exercise capacity of Group MT was significantly higher than that of Group PT (*p* < 0.05) but lower than that of Group E (*p* < 0.01). Correlation analysis showed that *Sutterella* abundance was negatively correlated with exercise capacity (*r* = −0.42, *p* < 0.05), whereas *Dorea* abundance was positively correlated (*r* = 0.48, *p* < 0.05).

**Conclusion:**

Exercise-induced increases in glycogen reserves and antioxidant capacity, associated with altered intestinal microbiota composition, can be partially transferred to recipient mice via FMT. The gut microbiota acts as a partial mediator of improvements in exercise capacity, rather than the sole driver.

## 1 Introduction

Improvements in exercise capacity arise via the synergistic effect of multiple mechanisms, such as metabolic regulation and antioxidant defense. Changes in the structure and function of the intestinal microbiota are closely related to host energy metabolism and immune homeostasis (Clarke et al., 2014; Scheiman et al., 2019; Clark and Mach, 2023; Zhang et al., 2024; Quaresma et al., 2024; Varghese et al., 2024; Guan and Liu, 2023). In this study, exercise capacity specifically refers to exhaustive running endurance in mice, quantified by the total distance covered during exhaustive running. In recent years, studies have shown that athletes’ intestinal microbiota exhibits unique functional characteristics. For example, the abundance of *Veillonella* in the feces of marathon runners is significantly increased; this bacterium can produce propionate by metabolizing lactic acid, and this metabolic process has been further verified in mouse models to significantly enhance exercise endurance (Scheiman et al., 2019). Metagenomic analysis of competitive cyclists has shown that the carbohydrate and amino acid metabolic pathways in their gut microbiota are significantly upregulated, and this functional characteristic is believed to be closely related to the demand for rapid energy supply during high-intensity cycling (Petersen et al., 2017). In addition, a comparative study of professional rugby players and the general physically active population has demonstrated that regular physical activity is associated with both increased gut microbial diversity and enriched microbial taxa related to energy metabolism and anti-inflammatory functions; moreover, changes in these microbial characteristics positively correlate with exercise intensity and duration (Barton et al., 2018). These findings suggest that the intestinal microbiota may be a key mediator of exercise-induced changes in host metabolism and exercise capacity. Longitudinal tracking of elite female football players has revealed that regular training during the season could maintain gut microbiota stability and reduce the abundance of harmful bacteria (e.g., *Desulfovibrio*) (Oliveira et al., 2022). In animal experiments, 8 weeks of moderate-intensity exercise increased the abundance of short-chain fatty acid-producing bacteria (e.g., *Faecalibaculum*) in the intestines of mice and improved microbiota metabolic function (Yuan et al., 2018). Collectively, these studies indicate the close association between physical activity and the gut microbiota from multiple dimensions, including population, sex, and species. In addition, in our previous study, we showed that moderate-intensity exercise can increase the abundance of *Prevotella*, a bacterium associated with enhanced carbohydrate metabolism, in the mouse intestinal tract (Yang et al., 2021). However, recent evidence indicates *Prevotella* may act as a confounding factor rather than a direct causal driver of exercise performance (Martin et al., 2026). Fecal microbiota transplantation (FMT) has been used to verify the regulatory effect of the gut microbiota on metabolic diseases (Yang et al., 2017; Heimesaat et al., 2019), and multiple rodent studies have shown that FMT can be used to explore microbiota-mediated exercise effects (Aoi et al., 2023; Lai et al., 2018; Martin et al., 2025). For example, Aoi W found that FMT from exercised mice enhanced glucose metabolism and exercise endurance in recipient mice (Aoi et al., 2023). Lai ZL showed that FMT altered the gut microbiota composition and enhanced antioxidant capacity in exercised mice (Lai et al., 2018), and Martin D further found that FMT from elite athletes increased skeletal muscle glycogen content in mice (Martin et al., 2025). However, it remains unclear whether exercise-induced microbiota changes can be directly transferred via FMT and improve glycogen storage, antioxidant capacity, and exercise capacity.

Building upon our previous work that evaluated the effect of exercise on intestinal microbiota composition (Yang et al., 2021), this study focuses on two core objectives: (1) to verify whether long-term endurance exercise-induced changes in the intestinal microbiota can be transferred to recipient mice via FMT and (2) to explore the correlation between specific microbiota genera and exercise capacity, providing supplementary evidence for the “exercise–microbiota–exercise capacity” regulatory axis. To address these questions, we subjected male C57BL/6 mice to a 14-week moderate-intensity treadmill exercise intervention [exercise group (Group E)] and transferred the intestinal microbiota to naïve mice [fecal microbiota transplantation group (Group MT)]; a group that did not engage in exercise (Group C) and a PBS transplantation group that received PBS instead of the fecal microbiota (Group PT) were included as controls. Next, the exhaustive exercise capacity of the mice, blood glucose and glycogen reserves, and antioxidant indices in the liver and muscles were detected, and the composition and function of the intestinal microbiota such as *α*/*β* diversity, differentially abundant genera, and Kyoto Encyclopedia of Genes and Genomes (KEGG) metabolic pathways were analyzed via 16S rRNA sequencing. This study provides scenario-specific evidence for the transferability of intestinal microbiota changes induced by long-term endurance exercise, and offers complementary *in vivo* evidence for the interactions among exercise, microbiota, and performance, supporting the use of microbiota as a target for regulating exercise capacity.

## 2 Materials and methods

### 2.1 Experimental animals

The experimental animals used in this study have been described previously in two earlier studies from our group (Yang et al., 2021; Yang et al., 2025). Fifty-two 5-week-old specific-pathogen-free-grade healthy male C57BL/6 mice, with an average body weight of 18 ± 2 g, were purchased from the Guangdong Provincial Medical Experimental Animal Center (SCXK (Guangdong) 2018–0002). The mice were housed three to four per cage at room temperature (20–25 °C), with 50 to 70% humidity, a 12/12-h light/dark cycle, with free access to food (basal diet purchased from the Guangdong Provincial Medical Experimental Animal Center) and water. All animal experiments were performed in accordance with the Guidelines for Ethical Review of Experimental Animals (GB/T 35892–2018) and approved by the Ethics Committee of the School of Sports Science, South China Normal University (approval number: SCNU-SPT-2019-002). After a 2-week acclimation period, the mice were randomly divided into four groups: the control group (Group C, *n* = 15): no exercise intervention; the long-term endurance exercise group (Group E, *n* = 15): 14 weeks of moderate-intensity treadmill exercise; the PBS transplantation group (Group PT, *n* = 11): intestinal lavage with antibiotics and polyethylene glycol, followed by transplantation with PBS; and the fecal microbiota transplantation group (Group MT, *n* = 11): intestinal lavage with antibiotics and polyethylene glycol, followed by transplantation with a fecal microbiota suspension from Group E.

The sample size was determined to ensure sufficient fecal material for FMT, with reference to previous studies (Yang et al., 2021; Yang et al., 2025). A total of 11 mice were included in each recipient group (PT/MT), and 15 mice in each donor/control group (E/C). This sample size minimizes individual variation and ensures adequate biological replicates for sequencing and biochemical analyses, balancing animal welfare and experimental costs. Only male mice were used to avoid estrous cycle interference with microbiota and metabolism.

### 2.2 Exercise intervention

The mice were first subjected to 1 week of low-intensity exercise to adapt to treadmill use, as follows: 3 days/week, 10–20 min/day, 0° incline, speed of 10–13 m/min. The formal exercise training lasted for 14 weeks and involved moderate-intensity treadmill exercise: 5 days/week, 60 min/day, 0° incline. The speed was set at 14 m/min for weeks 1 to 2, 15 m/min for weeks 3 to 6, 17 m/min for weeks 7 to 10, and 19 m/min for weeks 11 to 14 (Yang et al., 2021). Mice in all groups were housed under the same conditions to eliminate the influence of environmental factors (except for exercise) on the experimental results.

### 2.3 Fecal microbiota transplantation

#### 2.3.1 Intestinal clearance

Mice in Groups PT and MT were subjected to intestinal clearance with antibiotics and lavage with polyethylene glycol plus electrolytes (Yang et al., 2017; Heimesaat et al., 2019). First, the mice were treated with antibiotics (1 g/L metronidazole, 0.5 g/L vancomycin, 1 g/L neomycin sulfate, and 1 g/L ampicillin combined; Sigma-Aldrich) by gavage at a volume of 0.2 mL/mouse twice a day for 7 consecutive days. On day 8, no antibiotics were administered. Beginning on day 9, the mice were given an isotonic lavage solution instead of normal drinking water for 3 days. The lavage solution comprised polyethylene glycol 4,000 electrolyte powder II (sodium bisulfate, sodium chloride, anhydrous sodium sulfate, potassium chloride, and polyethylene glycol 4,000; Shenzhen Wanhua Pharmaceutical Co., Ltd.) dissolved in 1000 mL water to generate an isotonic whole-bowel irrigation solution with the following ion concentrations: 20 mmol/L HCO_3_^−^, 40 mmol/L SO_4_^2−^, 125 mmol/L Na^+^, 10 mmol/L K^+^, and 35 mmol/L Cl^−^.

Fecal samples were collected at three time points: pre-intestinal clearance (PT-Pre/MT-Pre), post-intestinal clearance (PT-Post/MT-Post), and post-FMT (PT-FMT/MT-FMT).

#### 2.3.2 Microbiota suspension preparation

At the 14th week of the exercise intervention, 15 mice from Group E (all with no obvious health abnormalities and no diarrhea/rapid weight loss) were selected as donors. These donor mice were each from different cages to avoid cage-effect interference. For each donor mouse, 50–100 mg of fresh feces was collected, and the fecal samples were mixed in equal proportion by weight (i.e., the feces from each donor mouse accounted for the same proportion of the total mixed sample) to form a single composite sample. Sterile normal saline was added to the combined fecal sample at a feces-to-saline ratio of 1:5, and the mixture was stirred for 30 min until it was homogeneous. The suspension was filtered through a 304 stainless steel funnel; the filtrate was transferred to a 15-mL centrifuge tube and centrifuged at 1500 rpm at 4 °C for 5 min, and the supernatant was discarded. Normal saline was added again at a 1:2 ratio based on the initial fecal weight. Subsequently, the above steps were repeated three times, followed by thorough mixing. The final microbiota suspension was mixed with sterile PBS at a 1:1 ratio, stored at 4 °C, and used within 30 min. The microbiota suspension was freshly prepared daily to ensure microbiota activity.

#### 2.3.3 Transplantation

On day 12, after the antibiotic treatment and intestinal lavage were complete, the mice were fasted for 1 day with free access to water. Beginning on day 13, they were subjected to FMT via enema daily for 7 days (Yang et al., 2017; Cammarota et al., 2017). First, defecation was stimulated by raising the mouse’s tail and gently rubbing the lower abdomen and perianal area. After defecation, the mice were anesthetized by being placed in a transparent plastic box containing a cotton ball soaked in isoflurane for approximately 3 s. To deliver the enema, the microbiota suspension (Group MT) or PBS (Group PT) was thoroughly mixed and aspirated into a 1-mL syringe connected to an enema tube (lubricated with sterile paraffin oil). The tube was inserted into the anus approximately 4 cm along the sacral curvature, and 0.2 mL of the solution was slowly injected. The syringe was then withdrawn. A cotton swab was pressed against the anus while lifting the mouse’s tail for 1 min to prevent fluid leakage. After transplantation, each mouse was monitored until it recovered from anesthesia and returned to its original cage after the absence of abnormalities was confirmed. FMT or PBS transplantation was performed twice a day (morning and evening) for 7 consecutive days to ensure stable microbiota colonization. Housing conditions during transplantation remained consistent with those before intervention to minimize interference from dietary differences on gut microbiota reconstruction.

### 2.4 Outcome measurements

#### 2.4.1 Intestinal microbiota analysis

Mice were placed in clean cages lined with sterile filter paper (one mouse per cage). The mouse’s tail was gently lifted to expose the anus, and the mouse was held upside down. A long cotton swab was used to gently rub the mouse’s lower abdomen and anus approximately 10 times to stimulate defecation. The mouse was then returned to the cage for defecation. The above actions were repeated until the mouse excreted approximately five fecal pellets. The feces were collected in sterile cryovials, immediately placed on dry ice, and stored at −80 °C after all samples were collected.

DNA was purified from the feces using SDS lysis buffer, as described previously (Yang et al., 2021), and stored at −20 °C. The extracted DNA was assessed using a NanoDrop ND-1000 spectrophotometer, followed by agarose gel electrophoresis. The V4 region of the 16S rRNA gene was PCR-amplified using forward primer 515F (5’-GTGCCAGCMGCCGCGGTAA-3’) and reverse primer 806R (5’-GGACTACHVGGGTWTCTAAT-3’). Barcode sequences and primer sequences were removed from the raw sequencing data. Chimeric sequences were removed from the raw tags to obtain the final effective tags. Inter-group comparisons of alpha diversity, beta diversity, and taxonomic abundance were performed, as well as LEfSe analysis, and microbial function prediction was conducted using PICRUSt. PICRUSt provides predictive inference; no direct metabolomic measurements (short-chain fatty acids (SCFAs), lactate, bile acids) were performed, as acknowledged in the limitations.

Operational taxonomic unit (OTU) clustering was performed on the V4 region sequences of the 16S rRNA gene using a 97% sequence similarity threshold (with UPARSE software) for subsequent microbiota composition and diversity analysis. The reasons for choosing the OTU method are as follows: (1) this study is a follow-up of the previous study, and the consistent OTU clustering method ensures data comparability, and (2) the study focuses on community changes at the genus level and above, and the resolution ability of the OTU method at this taxonomic level can meet the research needs.

To verify the engraftment efficiency of FMT, three donor-relative analyses were performed: (1) donor-recipient OTU overlap analysis: the number of shared OTUs and donor OTU coverage between donor Group E and recipient groups (PT-FMT, MT-FMT) were calculated and compared; (2) Bray–Curtis similarity analysis: global microbial similarity between donor Group E and recipient mice at three time points was evaluated; and (3) key genus tracking: the relative abundances of *Dorea*, *Sutterella*, and *Bifidobacterium* were quantified to assess donor-specific engraftment in recipient mice after FMT.

#### 2.4.2 Exhaustive exercise capacity test

Mice in groups PT and MT underwent exercise capacity tests before antibiotic treatment and polyethylene glycol intestinal lavage, as well as on day 20, 1 day after the final transplantation. Exercise capacity was tested as previously described (Yang et al., 2021). First, the mice were fasted for 2 h. Then, they were placed on a small-animal treadmill moving at a very low speed for 2 min to allow them to acclimate to the training environment, after which the treadmill speed was increased to 10 m/min (0° incline). The speed was increased by 1 m/min every minute until it reached 21 m/min at the 12th minute, and then maintained at 21 m/min from the 13th to the 120th minute. From the 121st minute, the speed was adjusted to 22 m/min, and then increased by 1 m/min every 20 min until exhaustion. The exhaustion time and speed were recorded, and the total running distance was calculated according to these. The criteria for determining mouse exhaustion were as follows: the mouse could not maintain the preset running speed, exhibited abnormal running posture, remained in the rear third of the treadmill after three consecutive attempts to move it forward for more than 10 s, showed rapid and irregular breathing and fatigue, and lay in a prone position with a drooping head after being removed from the treadmill. The maximum running distance was recorded as the mouse’s exhaustive exercise capacity (Zhong, 2015; Suzuki, 2025).

#### 2.4.3 Detection of biochemical indicators

##### 2.4.3.1 Group C (control group) and group E (long-term endurance exercise group)

At 48 h after the last exercise session at the completion of the long-term exercise intervention, when the mice were in a resting state, they were weighed and then sacrificed by an overdose of anesthesia: 1.25% avertin (2,2,2-tribromoethanol) was injected intraperitoneally at a dose of 0.6 mL/20 g of body weight. Blood was collected from the orbital sinus by removing the eyeball; the area over the heart was gently pressed with the thumb to increase blood collection volume, and the blood was allowed to drip into a collection tube. After standing at room temperature for 30 to 60 min, the blood was centrifuged at 2500 rpm at 4 °C for 15 min, and the serum was collected in an EP tube for later analysis. After the blood was collected, the abdominal cavity was opened, the right atrial appendage was cut, and perfusion was performed from the left ventricle using 4 °C normal saline to remove any remaining blood from the tissue. Then, the liver and gastrocnemius muscle were removed and stored at −80 °C. Serum insulin levels were determined using an insulin ELISA kit (Nanjing Jiancheng Bioengineering Institute) according to the kit instructions. Fasting blood glucose levels, hepatic glycogen content, and muscle glycogen content, MDA, T-AOC, and GSH levels, and SOD and CAT activities in the liver and gastrocnemius muscle were determined using a Roche blood glucose meter and ELISA kits (Nanjing Jiancheng Bioengineering Institute) according to the kit instructions.

##### 2.4.3.2 Group PT (PBS transplantation group) and group MT (fecal microbiota transplantation group)

On the day after the final transplantation (i.e., Day 20), fecal samples were collected from the mice (using the same method as described in 2.4.1 Intestinal Microbiota Analysis). Subsequently, the mice underwent the exhaustive exercise test (following the protocol outlined in 2.4.2 Exhaustive Exercise Capacity Test). Immediately after the exercise, the mice were weighed and sacrificed using the same overdose anesthesia method described above (1.25% avertin, intraperitoneal injection at 0.6 mL/20 g of body weight). No biochemical indicator detection was performed at this stage.

Glycogen and insulin levels in the PT and MT groups were not measured at rest. This study forms part of a sequential research project focusing on intestinal immune function after acute exhaustive exercise (Martin et al., 2026); thus, tissue sampling was unified to post-exercise. Related resting-state metabolic indicators will be supplemented in future studies.

### 2.5 Statistical analyses

Statistical analyses were performed using SPSS 20.0 software and R packages (v3.2.0), and QIIME software (v1.9.0) was used to process microbial community sequencing data. The data are presented as the mean ± standard deviation (mean ± SD).

(1) Data Preprocessing: The Shapiro–Wilk test was used to assess data normality. Parametric tests were applied to normally distributed data, whereas non-parametric tests were used for non-normally distributed data.
(2) Inter-group Comparisons: Inter-group comparison of host biochemical indicators (Control Group C *vs.* Exercise Group E): Since the data conformed to a normal distribution, an independent-samples *t* test was used to evaluate inter-group differences. For exhaustive exercise capacity indicators among multiple groups, one-way analysis of variance (ANOVA) was performed for inter-group statistical comparison. Microbial community analysis: In the analysis of taxonomic units (from phylum to genus level) and functional pathways, the Benjamini–Hochberg (BH) method was employed for multiple-comparison correction to control the false discovery rate (FDR). *β*-diversity analysis was conducted using the Bray–Curtis and weighted UniFrac distance. The PERMANOVA test (permutation number = 999, implemented via the R package “vegan”) was used to assess the explanatory power of grouping on variations in community structure. Linear discriminant analysis effect size (LEfSe) was used to screen for inter-group differential taxonomic units, with the linear discriminant analysis (LDA) cutoff value set at 2.0. Only taxonomic units with *p* < 0.05 (after a Kruskal–Wallis test for overall multi-group differences and a Wilcoxon test for pairwise differences) were considered significant differential taxa.
(3) Sequencing Data Processing: Raw sequencing reads were subjected to quality filtering to remove low-quality sequences (sequence length < 150 bp, average Phred quality score < 20, sequences containing ambiguous bases or mononucleotide repeats > 8 bp) and chimeric sequences (using Vsearch v2.4.4 software), resulting in the acquisition of effective tags. OTUs were clustered from the effective tags at a 97% sequence similarity threshold (via Vsearch v2.4.4 software). Representative sequences of OTUs were aligned against the SILVA128 database for taxonomic annotation. OTUs accounting for < 0.001% of the total sequences across all samples were removed to reduce data noise. Rarefaction normalization was performed by rarefying all samples to the minimum sequencing depth, ensuring comparability between samples in the *α*-and *β*-diversity analyses.
(4) Significance judgment: *p* < 0.05 was considered to indicate significance. *Post hoc* power analysis was performed for primary endpoints. The exhaustive exercise capacity (PT-FMT *vs.* MT-FMT) achieved a power of 0.800. The Shannon index and *Firmicutes* abundance reached power values of 0.993 and 0.830, respectively. All significant results were based on adequate statistical power (≥ 0.8) and large effect sizes.

## 3 Results

We previously demonstrated that the intestinal microbiota in Group E is enriched in metabolic pathways compared with that of Group C, including glucose metabolism, flavonoid metabolism, arginine metabolism, and proline metabolism (Yang et al., 2021). Here, we further analyzed metabolism-related indicators and microbiota transferability.

### 3.1 Long-term endurance exercise increases hepatic and muscle glycogen levels and superoxide dismutase activity in mice

To determine whether long-term endurance exercise alters energy production and antioxidant activity in mice, we measured indicators of both processes in the blood, liver, and gastrocnemius muscles of mice subjected to exercise and control (non-exercised) mice. As shown in Table 1, there were no significant differences in fasting blood glucose and insulin levels between Group E (exercise group) and Group C (control group). The mean hepatic glycogen level in Group E was significantly higher than that in Group C (*p* < 0.01), and the mean muscle glycogen content in Group E was significantly higher than that in Group C (*p* < 0.05).

**Table 1:** Biochemical indicator levels (mean ± SD).

Group (*n* = 8)		C	E
Blood glucose (mmol/L)		5.76 ± 1.1	5.25 ± 0.77
Insulin (nIU/ml)		25.21 ± 8.78	16.17 ± 9.03
Liver	Glycogen (mg/g)	11.54 ± 1.55	13.6 ± 1.08**
Gastrocnemius muscle		4 ± 1.54	5.32 ± 0.73*
Liver	MDA (nmol/mg protein)	2.61 ± 0.58	1.53 ± 0.69*
	T-AOC (mM Trolox)	0.36 ± 0.05	0.4 ± 0.08
	GSH (μmol/g protein)	11.4 ± 2.23	12.51 ± 1.31
	CAT (U/mg protein)	1.69 ± 0.86	2.09 ± 0.93
	SOD (U/mg protein)	34.91 ± 3.31	39.93 ± 4.15**
Gastrocnemius muscle	MDA (nmol/mg protein)	2.32 ± 0.59	1.66 ± 0.98
	T-AOC (mM Trolox)	0.29 ± 0.07	0.37 ± 0.08
	GSH (μmol/g protein)	10.63 ± 1.14	11.45 ± 2.2
	CAT (U/mg protein)	1.46 ± 0.87	1.77 ± 0.9
	SOD (U/mg protein)	36.23 ± 3.94	41.24 ± 2.5*

Control group (C): no exercise intervention; long-term endurance exercise group (E): 14-week moderate-intensity treadmill exercise. Statistical differences were assessed via an independent-samples t test. *p < 0.05, **p < 0.01 vs. group C. MDA, malondialdehyde; T-AOC, total antioxidant capacity; GSH, glutathione; CAT, catalase; SOD, superoxide dismutase.

As shown in Table 1, the mean liver MDA level in Group E was significantly lower than that in Group C (*p* < 0.05), and the mean liver SOD activity in Group E was significantly higher than that in Group C (*p* < 0.01). There were no significant differences in mean liver T-AOC, GSH level, or CAT activity between Groups E and C.

The mean gastrocnemius SOD activity in Group E was significantly higher than that in Group C (*p* < 0.05; Table 1). There were no significant differences in mean gastrocnemius T-AOC, GSH level, or CAT activity between Groups E and C.

Taken together, these results indicate that long-term endurance exercise improves energy storage and antioxidant defense in mice.

### 3.2 Fecal microbiota transplantation from mice subjected to long-term endurance exercise alters the intestinal microbiota composition of recipient mice

#### 3.2.1 Sequencing results and quality control

To determine whether and how FMT from mice subjected to long-term endurance exercise alters the intestinal microbiota of recipient mice, we collected fecal samples at three time points: pre-intestinal clearance, post-intestinal clearance, and post-FMT. Fecal samples collected from Group PT at these three time points were denoted PT-Pre, PT-Post, and PT-FMT, respectively; those collected from Group MT were denoted MT-Pre, MT-Post, and MT-FMT, respectively. The numbers of usable sequences (tags), clean tags, and OTUs obtained for Groups PT and MT at all three time points are shown in the Supplementary Information (Supplementary Table 1). Comparing the OTUs for each group and time point showed the presence of shared and unique OTUs (Supplementary Figure 1).

To assess the diversity of the intestinal microbiota in both groups of recipient mice at all three timepoints, we calculated the Shannon diversity index and plotted it against sequencing depth. As shown in Supplementary Figure 2, the number of OTUs and Shannon diversity increased significantly with increasing sequencing depth at all three time points for Groups PT and MT and failed to plateau, indicating that new bacterial species might be found with further increases in sequencing depth. However, as the sequencing depth gradually increased, all Shannon–Winner curves reached a plateau, indicating that the bacterial species in the samples were fully represented at this sequencing level.

Taken together, these findings indicate that we accurately detected comprehensive changes in the intestinal microbiota in both groups at all time points.

#### 3.2.2 Fecal microbiota transplantation alters microbial abundance at the phylum and genus levels

**Figure 1:**
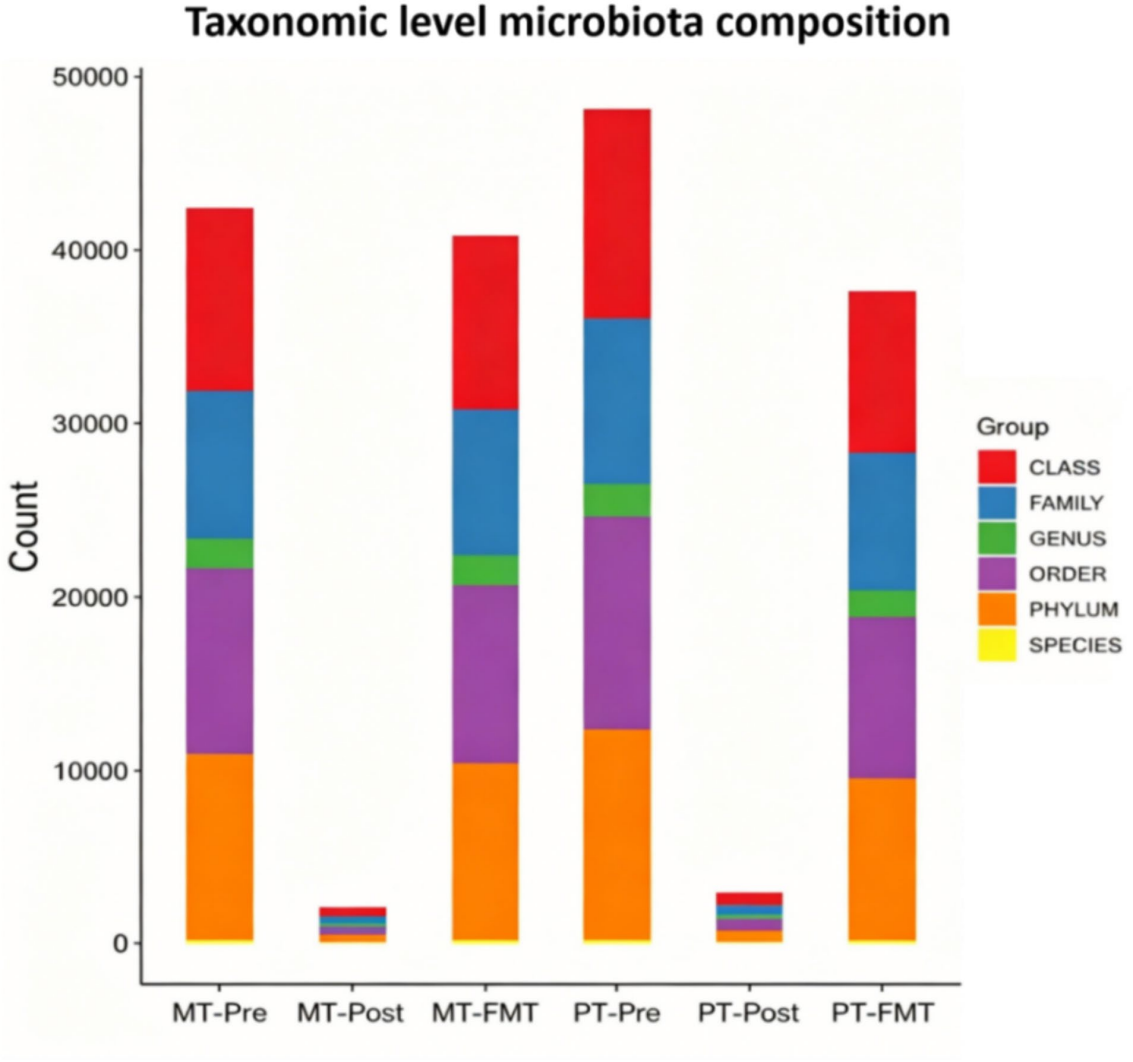
Number of sequences obtained at different taxonomic levels for groups PT and MT at the three time points. The y-axis represents the number of sequences, and the x-axis represents the group.

To determine which microbial taxa were most profoundly affected by fecal microbiota transplantation from exercised and non-exercised mice, we compared microbial counts across taxonomic levels for both groups at all three time points. As shown in Figure 1, the numbers of sequences at all taxonomic levels in PT-Post and MT-Post were significantly decreased compared with those at PT-Pre and PT-FMT and MT-Pre and MT-FMT, respectively, indicating successful clearance of the native microbiota. FMT effectively restored microbe counts in the MT-FMT group; to a lesser extent, PBS transplantation restored counts in the PT-FMT group, likely attributable to colonization by microbes ingested by the mice through their food and drinking water.

**Figure 2:**
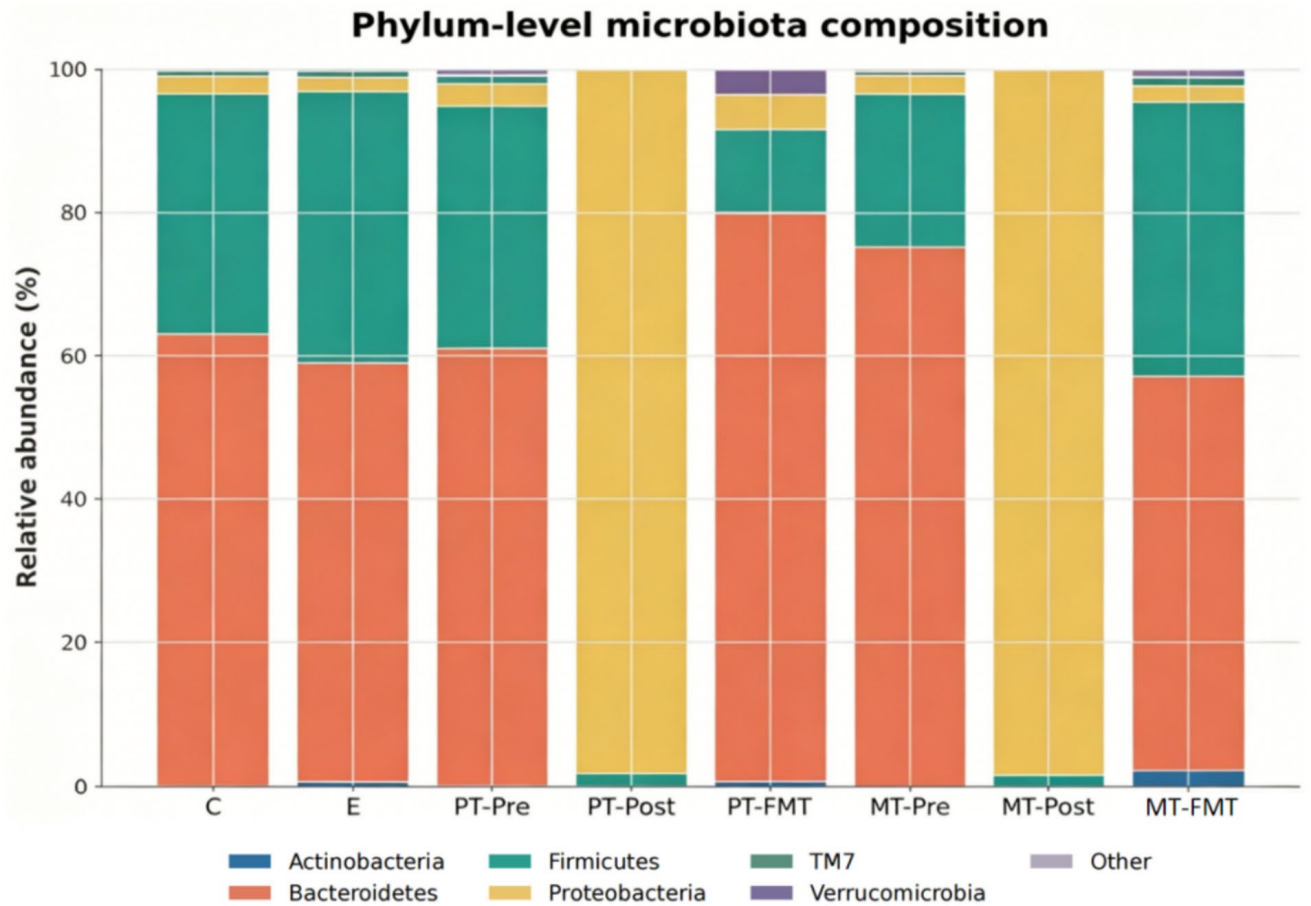
Phylum-level microbiota composition. The relative abundance of Proteobacteria increased after intestinal clearance, and the community structure was reshaped after transplantation, showing distinct compositions between MT-FMT and PT-FMT.

As shown in Figure 2, the most abundant phylum in PT-Pre and MT-Pre was *Bacteroidetes*, accounting for 60.81 and 75.18% of reads, respectively, followed by *Firmicutes* (34.22 and 21.48%, respectively) and *Proteobacteria* (2.94 and 2.53%, respectively). Together, these three phyla accounted for 97.97 and 99.20% of the reads obtained for PT-Pre and MT-Pre, respectively. The most abundant phylum in PT-FMT and MT-FMT was *Bacteroidetes*, accounting for 79.20 and 55.20% of reads, respectively, followed by *Firmicutes* (11.95 and 38.38%, respectively) and *Proteobacteria* (4.80 and 2.21%, respectively). Together, these three phyla accounted for 95.96 and 95.80% of the reads obtained for PT-FMT and MT-FMT, respectively.

At the phylum level, the relative abundances of *Firmicutes*, *TM7*, and *Tenericutes* in MT-FMT were significantly higher than those in PT-FMT (*p* < 0.01). The relative abundances of *Bacteroidetes* and *Proteobacteria* in MT-FMT were significantly lower than those in PT-FMT (*p* < 0.05). The *Firmicutes*/*Bacteroidetes* (F/B) ratio in MT-FMT was significantly higher than that in PT-FMT (*p* < 0.01).

There were 12 differentially abundant genera between PT-FMT and MT-FMT. MT-FMT was significantly enriched in eight beneficial genera (*Bifidobacterium*, *Adlercreutzia*, *Paraprevotella*, *Clostridium*, *Dorea*, *Oscillospira*, *Ruminococcus*, and *Allobaculum*) compared with PT-FMT (*p* < 0.05). MT-FMT exhibited significantly lower abundances of two harmful/conditional genera (*Parabacteroides* and *Sutterella*) than PT-FMT (*p* < 0.01).

Taken together, these results indicate that FMT versus PBS transplantation dramatically altered the intestinal microbiota composition of recipient mice at the phylum and genus levels, with FMT enhancing the abundance of beneficial microbes and decreasing the abundance of harmful microbes.

#### 3.2.3 Fecal microbiota transplantation results in increased microbial diversity compared with phosphate-buffered saline transplantation

**Figure 3:**
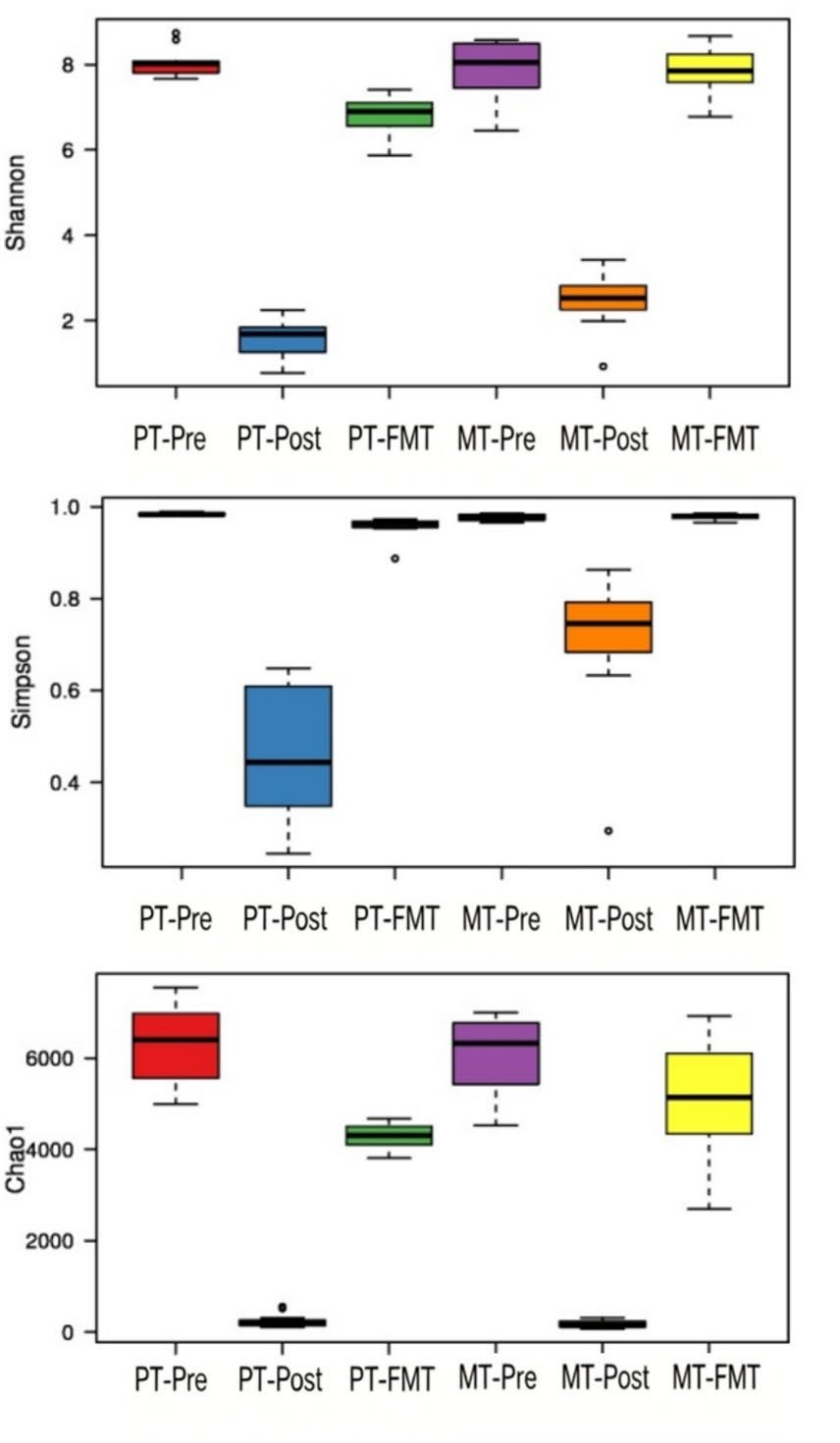
Boxplots of *α*-diversity index differences for groups PT and MT at different time points. The panels show the Shannon, Simpson, and Chao1 indices. The x-axis shows the group name, and the y-axis shows the alpha diversity index for each group. The boxplots display five values for each condition (i.e., minimum, first quartile, median, third quartile, and maximum, from bottom to top). Outliers are marked with “o.” Differences in alpha diversity among the six groups (PT1, PT2, PT3, MT1, MT2, MT3) were determined by Kruskal–Wallis test (overall *p* < 0.01). Pairwise comparisons were performed using the Wilcoxon rank-sum test with FDR correction.

To assess the diversity of the intestinal microbiota of recipient mice after transplantation, we calculated *α*- and *β*-diversity indices at each time point. For α diversity, the Shannon, Simpson, and Chao1 indices were calculated for each group (Figure 3), with all values significantly lower in the PT-Post and MT-Post groups than in the PT-Pre, MT-Pre, PT-FMT, and MT-FMT groups. The Shannon and Simpson index values for MT-FMT were significantly higher than those for PT-FMT (*p* < 0.01), and there was no significant difference in the Chao1 index values between the two groups. Pairwise comparisons between groups are presented in Supplementary Table 2.

**Figure 4:**
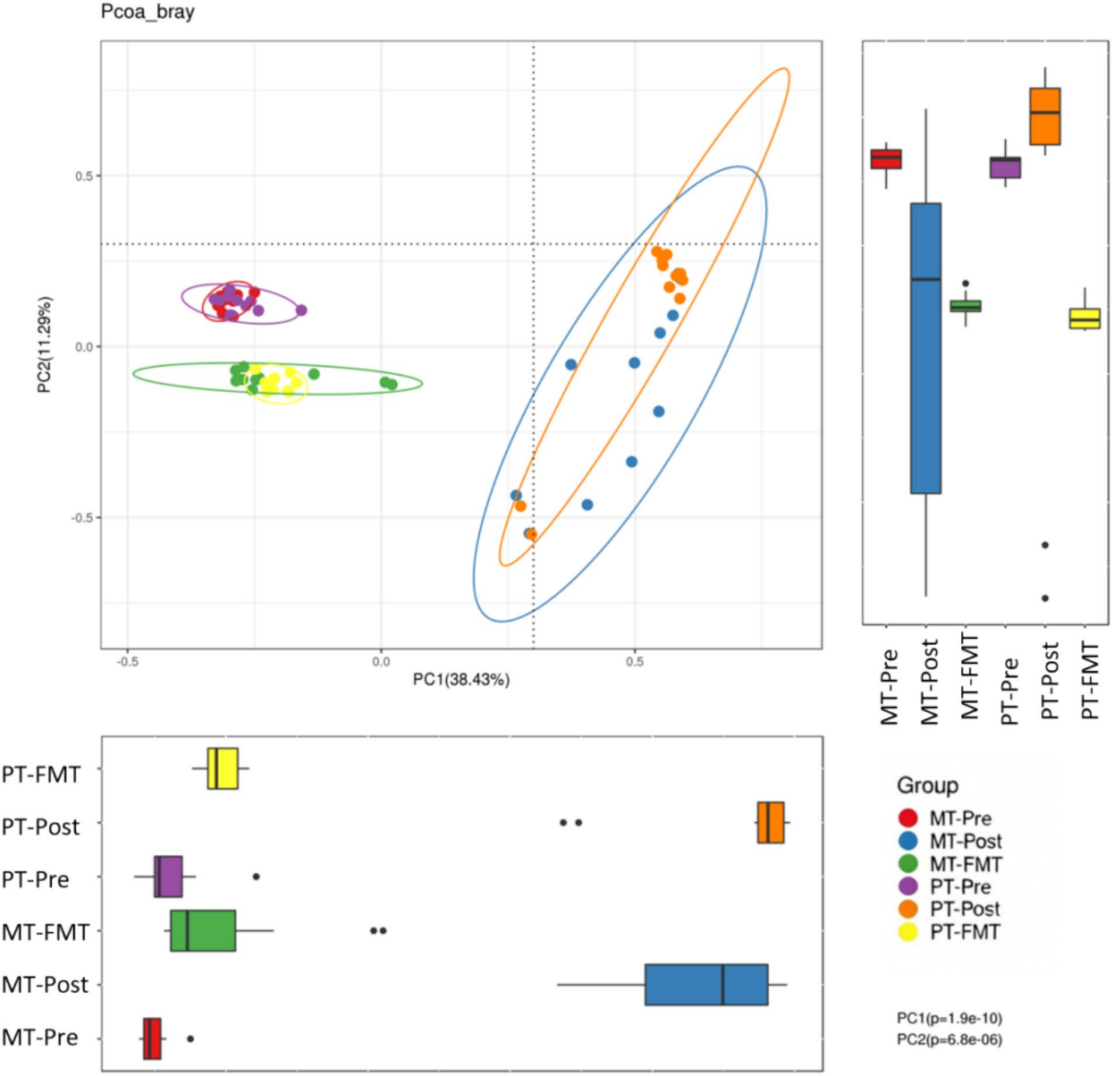
Principal coordinate analysis (PCoA) of the gut microbiota based on Bray–Curtis distance for groups PT and MT at different time points. Each point represents a single sample, with points of the same color belonging to the same group, and their distance reflecting the degree of similarity in microbial community composition. *β*-diversity analysis was conducted using both the Bray–Curtis and weighted UniFrac distances. The PERMANOVA test (permutation number = 999, implemented via the R package “vegan”) was used to assess the explanatory power of grouping on variations in community structure. The percentages of variation explained by the first two principal coordinates are shown on the axes (PC1: 38.43%, PC2: 11.29%), with the corresponding *p* values for group differences noted as PC1 (*p* = 1.9e−10) and PC2 (*p* = 6.8e−06).

To assess β diversity, we performed PCoA analysis based on Bray–Curtis distance. As shown in Figure 4, there were significant differences in β diversity across the six groups (PT-Pre, PT-Post, PT-FMT, MT-Pre, MT-Post, and MT-FMT; *p* < 0.01). There was a significant difference in β diversity between PT-FMT and MT-FMT (*p* < 0.01). The similar Bray–Curtis distance across groups pre-FMT may be attributable to uniform intestinal clearance, and the post-FMT differences confirm microbiota restructuring.

Taken together, these data show that FMT led to greater intestinal microbiota diversity in recipient mice than did PBS transplantation.

### 3.3 Fecal microbiota transplantation alters metabolic pathway activity in mice

To determine the functional effects of the changes observed in the intestinal microbiota of FMT-recipient mice, we performed PICRUSt analysis to identify differentially active metabolic pathways between groups. Forty level-three KEGG metabolic pathways showed significant differences between PT-FMT and MT-FMT; 7 key pathways are shown in Table 2. Specifically, the flavone and flavonol biosynthesis pathway, starch and sucrose metabolism pathway, and alpha-linolenic acid metabolism pathway are directly related to exercise capacity; the flagellar assembly pathway and bacterial chemotaxis pathway are associated with microbial colonization and intestinal mucosal barrier function; and the ether lipid metabolism pathway and dioxin degradation pathway are related to antioxidant defense and detoxification. All 7 pathways exhibited significantly higher activities in MT-FMT versus PT-FMT.

**Table 2:** Differentially active level-three KEGG metabolic pathways between PT-FMT and MT-FMT.

Pathway	PT-FMT (%)	MT-FMT (%)
Bacterial chemotaxis	2.48 ± 3.36	6.61 ± 7.25*
Flagellar assembly	1.88 ± 1.66	7.21 ± 8.21*
Dioxin degradation	2.11 ± 1.12	6.98 ± 6.89**
Ether lipid metabolism	0.42 ± 0.3	8.67 ± 11.49**
Flavone and flavonol biosynthesis	2.14 ± 1.84	6.95 ± 7.56*
Starch and sucrose metabolism	3.77 ± 5.72	5.32 ± 4.76*
Alpha-linolenic acid metabolism	1.78 ± 2.41	7.31 ± 10.49*

PBS transplantation group (PT) and fecal microbiota transplantation group (MT) were defined as PT-FMT and MT-FMT after transplantation. Statistical differences were assessed via the Wilcoxon test. *p < 0.05, **p < 0.01 vs. PT-FMT.

### 3.4 Fecal microbiota transplantation from mice subjected to long-term endurance exercise increases exercise capacity in recipient mice

To determine whether the changes in intestinal microbiota composition and metabolic pathway activation after FMT correlate with functional changes in exercise performance, we tested the exhaustive exercise capacity of mice in the MT and PT groups at all three time points. As shown in Table 3, there was no significant difference in exhaustive exercise capacity between the PT and MT groups before intestinal lavage (PT-Pre, MT-Pre). After transplantation, the exhaustive exercise capacity of mice in Group MT was significantly higher than that of mice in Group PT (*p* < 0.05). In addition, the exhaustive exercise capacity of mice in both groups after transplantation was significantly lower than that of mice in Group E (*p* < 0.01), while that of mice in Group MT after transplantation was significantly higher than that of mice in Group C (*p* < 0.01). There was no significant difference between the exhaustive exercise capacity of mice in Group PT after transplantation and mice in Group C.

**Table 3:** Exhaustive exercise capacity of mice.

Group	Exhaustive exercise capacity (m)
C	1441.27 ± 346.48
E	4274.29 ± 631.48**
PT-Pre	1247.45 ± 661.71
MT-Pre	936.27 ± 321.77
PT-FMT	1977.36 ± 909.72##
MT-FMT	2,990 ± 892.54**##$

Control group (C): no exercise intervention; long-term endurance exercise group (E): 14-week moderate-intensity treadmill exercise. The PBS transplantation group (PT) and fecal microbiota transplantation group (MT) were designated as PT-Pre and MT-Pre before intestinal clearance, and defined as PT-FMT and MT-FMT after transplantation. Statistical differences were analyzed by one-way analysis of variance (ANOVA). **p < 0.01 vs. Group C; ##*p* < 0.01 vs. Group E; $p < 0.05 vs. Group PT-FMT.

Next, we performed a correlation analysis to assess the association between differentially abundant genera and exercise capacity. The genus *Sutterella*, which was differentially abundant between MT-FMT and PT-FMT, was negatively correlated with exercise capacity (*r* = −0.42, *p* < 0.05), whereas *Dorea* was positively correlated (*r* = 0.48, *p* < 0.05).

Taken together, these findings indicate that exercise-induced changes in the mouse intestinal microbiota associated with increased exercise capacity can be partially transferred to recipient mice via FMT (the exercise capacity of the MT-FMT group was significantly higher than that of the PT-FMT group but lower than that of the Group E), indicating an association between the intestinal microbiota and increased exercise capacity. Furthermore, the intestinal microbiota can partially mediate improvements in exercise capacity; however, FMT failed to fully recapitulate the exercise capacity phenotype of Group E, suggesting that the regulation of exercise capacity by exercise is the result of a synergistic effect of multiple factors. Thus, the intestinal microbiota is not the sole driver.

### 3.5 FMT colonization efficiency verification

Next, we verified the colonization effect of fecal microbiota from long-term endurance exercise mice (Group E as donor) on recipient mice by comparing the intestinal microbiota characteristics of the PT and MT groups at different stages. Both groups of recipients underwent three stages: PT-Pre/MT-Pre, PT-Post/MT-Post, and PT-FMT/MT-FMT. Specifically, the PT-FMT group received PBS transplantation, whereas the MT-FMT group received fecal microbiota transplantation from Group E. The findings are detailed below.

#### 3.5.1 Global microbiota recovery: reconstruction after clearance, no significant difference between MT-FMT and PT-FMT after transplantation

**Figure 5:**
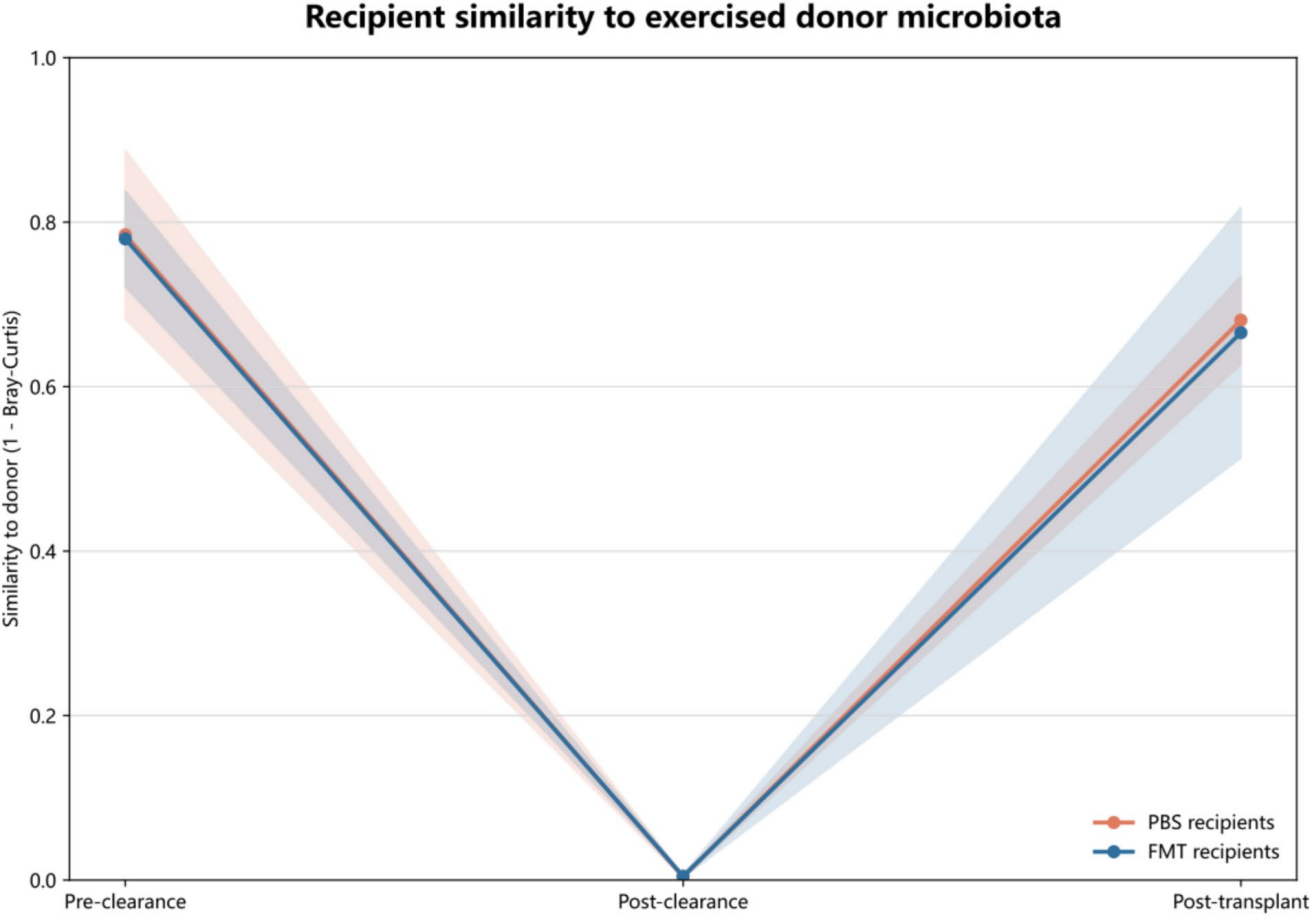
Bray–Curtis similarity of recipient gut microbiota to exercise donors. Both PT and MT groups showed a significant decrease after clearance, followed by an increase after transplantation; this index supports community reconstitution but is insufficient to independently confirm donor-specific colonization.

The Bray–Curtis similarity index was used to analyze the overall structural correlation between the recipient and donor microbiota. After intestinal clearance (PT-Post/MT-Post), the similarity between both groups and the donor dropped to nearly 0, indicating that the original intestinal microbiota was effectively cleared. After transplantation (PT-FMT/MT-FMT), the similarity of both groups rebounded (average similarity of MT-FMT: 0.666; average similarity of PT-FMT: 0.681); however, there was no significant difference between the two groups (*p* = 0.393; Figure 5). This finding indicates that transplantation can promote the overall recovery of intestinal microbiota; however, the global similarity reflects only a trend in community reconstruction and cannot definitively confirm colonization by donor-specific microbiota.

#### 3.5.2 Donor OTU retention: MT-FMT retains more donor-specific fragments

**Figure 6:**
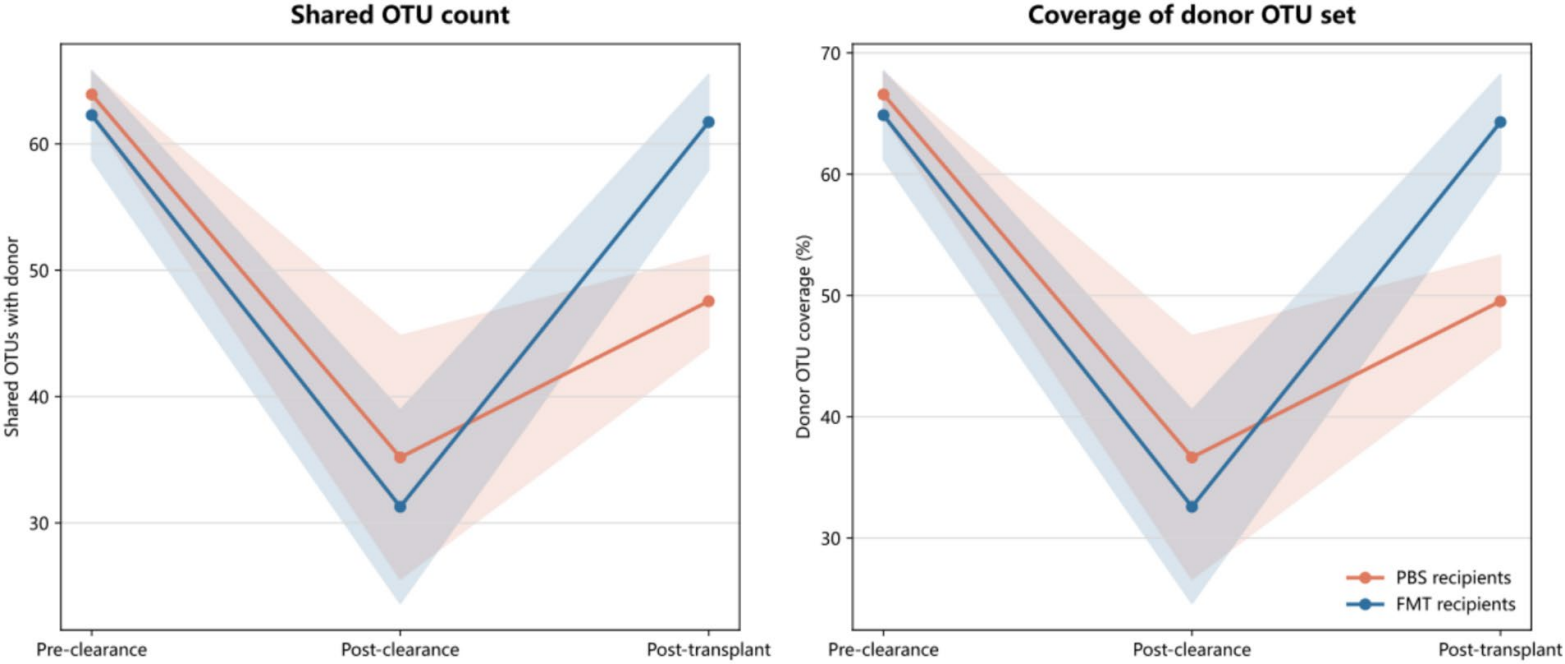
Shared OTU indicators between donors and recipients. MT-FMT showed higher values than PT-FMT in both the number of donor-shared OTUs and donor OTU coverage, indicating that the exercise donor microbiota left a stronger donor imprint in MT-FMT.

The ability to retain donor OTUs was compared between the two groups after transplantation: Number of shared OTUs: The average number of shared OTUs between MT-FMT and the donor was 61.73, which was significantly higher than the 47.55 shared OTUs between PT-FMT and the donor. Donor OTU coverage: The donor OTU coverage of MT-FMT reached 64.30%, which was significantly higher than the 49.53% of PT-FMT (both *p* = 7.64 × 10^−5^; Figure 6). In addition, the similarity between MT-FMT and the donor’s core microbiota, as well as the OTU overlap rate of MT-FMT with the donor, were both higher than those of PT-FMT (*p* < 0.05; Table 4). These results confirm that MT-FMT retains a greater proportion of donor-specific OTU fragments.

**Table 4:** Direct OTU comparison of MT-FMT/PT-FMT with donor group E.

Metric	PT-FMT	MT-FMT
Raw OTU Bray–Curtis similarity to E-donor centroid	0.158 ± 0.024	0.180 ± 0.035*
Pairwise donor OTU overlap with E group (%)	23.89 ± 2.03	30.46 ± 5.74*
Pairwise OTU Jaccard overlap with E group (%)	11.90 ± 0.86	13.47 ± 1.01**

The Bray–Curtis index is based on the similarity between recipient samples and the E-donor centroid in the raw OTU table. The shared OTU ratio and Jaccard index are calculated as the mean values obtained after pairwise comparisons between each recipient sample and every E donor sample. The p value is derived from a sample-level Mann–Whitney test comparing the PT-FMT and MT-FMT groups, *p < 0.05, **p < 0.01 vs. PT-FMT.

#### 3.5.3 Dynamics of key genera: MT-FMT is closer to donor genus characteristics

**Figure 7:**
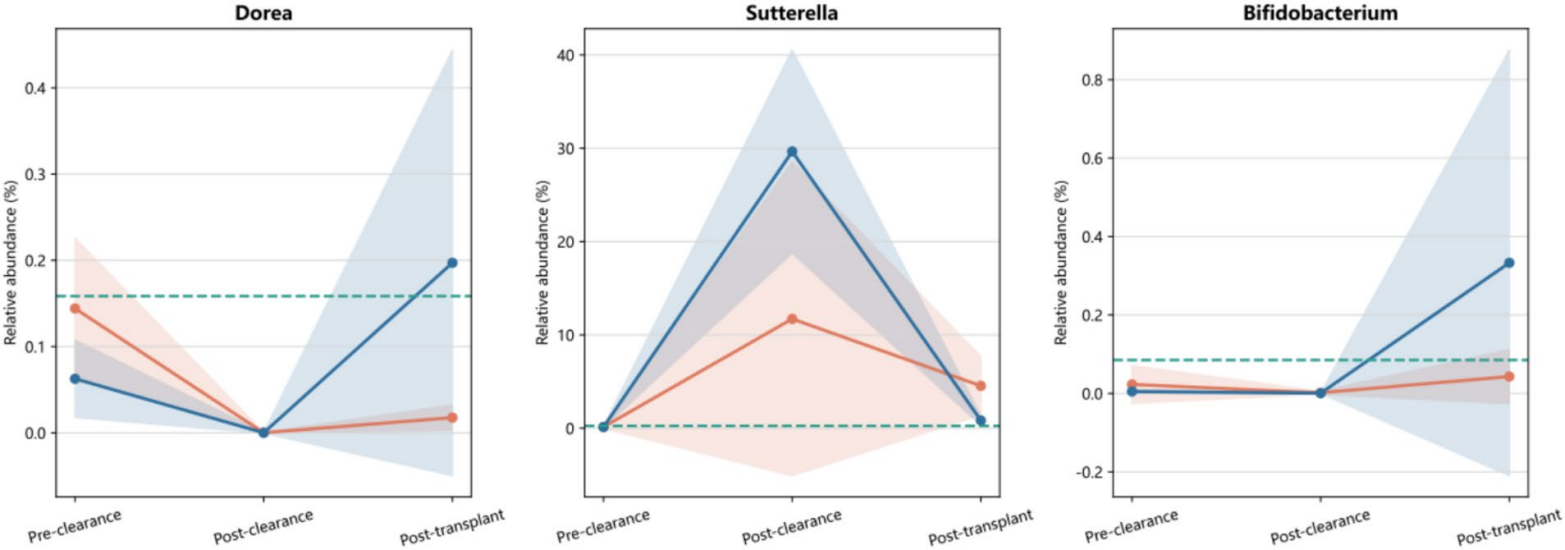
Dynamics and relative abundance of key genera before and after transplantation. The trajectory of the MT group was closer to the characteristics of the exercise donor (i.e., higher abundance of *Dorea* and *Bifidobacterium*, and lower abundance of *Sutterella*) after transplantation, indicating that donor-specific genus signatures were preserved in MT-FMT. Statistical analysis was performed using the Mann–Whitney U test. Compared with PT-FMT, MT-FMT showed significantly higher relative abundances of *Dorea* (*p =* 0.00252) and *Bifidobacterium* (*p* = 0.0151), and significantly lower abundance of *Sutterella* (*p* = 8.15e−05).

As shown in Table 5 and Figure 7, three key genera found to be closely associated with the donor microbiota were selected for subsequent analysis.

**Table 5:** Key indicators for FMT colonization efficacy (PT-FMT vs. MT-FMT).

Metric	PT-FMT	MT-FMT
Donor Bray–Curtis similarity	0.681 ± 0.053	0.666 ± 0.152
Shared OTU count with donor	47.55 ± 3.64	61.73 ± 3.77**
Donor OTU coverage (%)	49.53 ± 3.79	64.30 ± 3.93**
Dorea abundance (%)	0.018 ± 0.015	0.197 ± 0.247**
*Sutterella* abundance (%)	4.544 ± 3.136	0.827 ± 0.500**
*Bifidobacterium* abundance (%)	0.043 ± 0.069	0.333 ± 0.543*

The *p* value was obtained from a sample-level Mann–Whitney test between the PT-FMT and MT-FMT groups. Bray–Curtis similarity reflects global community restoration, while donor OTU coverage and the dynamics of key genera reflect the donor-specific imprint. *p < 0.05, **p < 0.01 vs. PT-FMT.

Positively associated genera (*Dorea*, *Bifidobacterium*): The relative abundances of *Dorea* and *Bifidobacterium* in MT-FMT were 0.197 and 0.333%, respectively, which were significantly higher than those in PT-FMT (0.018 and 0.043%, respectively). Negatively associated genus (*Sutterella*): The relative abundance of *Sutterella* in MT-FMT was 0.827%, which was significantly lower than the 4.544% in PT-FMT (all *p* < 0.05). Moreover, the genus abundances in MT-FMT were closer to those in the donor Group E, further indicating that the donor microbiota successfully colonized in MT-FMT.

The fecal microbiota from exercised mice achieved “partially effective colonization” in the MT-FMT group: Transplantation promoted microbiota recovery, and the MT-FMT group retained more donor OTUs (e.g., the number of OTUs shared with the donor was significantly higher than that in the PT-FMT group) and donor-specific genus characteristics (e.g., a higher abundance of *Dorea* and *Bifidobacterium* and a lower abundance of *Sutterella*); however, notably, there was no significant difference in Bray–Curtis similarity (reflecting overall community similarity) between the PT-FMT and MT-FMT groups, indicating that the donor microbiota only achieved partial feature engraftment rather than complete colonization of the entire community.

## 4 Discussion

In this study we found that long-term endurance exercise in mice increased hepatic and muscle glycogen levels and SOD activity and reduced liver MDA levels compared with the controls, indicating that long-term endurance exercise can improve exercise-related metabolic functions in mice by enhancing glycogen synthesis capacity and the antioxidant defense system. Furthermore, we showed that improvement in the exhaustive exercise capacity of mice may be related to changes in core intestinal bacteria that promote glycogen synthesis capacity and antioxidant function. Finally, transplanting the intestinal microbiota of mice subjected to exercise training significantly changed the intestinal microbiota structure of recipient mice (e.g., by increasing *Firmicutes* abundance and *α* diversity) and improved their exhaustive exercise capacity; however, the effect was not as strong as that observed with a direct exercise intervention.

The increase in exercise capacity observed in Group E and Group MT may be attributable to enhanced glycogen production and increased antioxidant function, consistent with our previously reported microbiota function data (Yang et al., 2021). Glycogen, as the main energy source during exercise, directly affects exercise endurance (Clarke et al., 2014; Scheiman et al., 2019); thus, the increase in glycogen levels seen in Group E may be related to exercise-induced increases in insulin sensitivity and glycogen synthase activity. Although we did not observe a significant change in insulin levels in Group E, previous studies have shown that long-term exercise can increase activation of the insulin signaling pathway, promote glucose transporter 4 (GLUT4) translocation to the cell membrane, and accelerate glucose uptake and glycogen synthesis (Richter and Hargreaves, 2013; Sylow et al., 2016). Although liver MDA levels were significantly reduced in Group E compared with those in Group C, there was no significant change in gastrocnemius MDA levels, which may be related to differing responses of different tissues to exercise-induced oxidative stress. The liver is the metabolic center of the body and the main source of energy during exercise; thus, its lipid peroxidation level is more likely to be regulated by exercise (Groussard et al., 2019; Yang et al., 2023). The aforementioned findings highlight the key roles of glycogen and antioxidants in increasing exercise capacity.

Our finding that moderate-intensity endurance exercise improved the exhaustive exercise capacity of mice, possibly through changes in core intestinal bacteria, is consistent with studies showing that the abundance of certain bacteria is closely related to metabolism. For example, one study found that an increase in *Prevotella* abundance is associated with the amount of weekly exercise and the activation of carbohydrate and amino acid metabolic pathways (Petersen et al., 2017). Another showed that the metabolic pathways of professional International Rugby Union players are relatively enhanced compared with those in the control group, including carbohydrate and amino acid metabolism pathways, which may be related to overall improved health (Clarke et al., 2014). Another study demonstrated that in top marathon runners, *Veillonella* abundance in the feces was significantly higher after a race than before. *Veillonella* can break down lactic acid to obtain carbon sources required for its own growth and produces propionate as part of this process. Delivering propionate to mice via an enema improved their heart rate and oxygen utilization in the absence of exercise training and also promoted metabolism in human subjects. Delivering *Veillonella* to mice by gavage also significantly increased their exercise endurance, consistent with the effect of the propionate enema (Scheiman et al., 2019). Furthermore, studies have demonstrated that the antioxidant enzyme system can prevent oxidative damage caused by strenuous exercise and is related to athletes’ physical condition. The intestinal microbiota may be an important factor in the host’s antioxidant defense (Hsu et al., 2015). Thus, the state of the intestinal microbiota may affect the host’s endogenous antioxidant enzyme system, and both the intestinal microbiota and the endogenous antioxidant enzyme system appear crucial for athletes’ performance. Thus, our findings suggest that exercise may alter the abundance of certain bacteria in the intestine and related metabolic pathways, thereby enhancing physical performance.

Our observation that the abundance of specific taxa is correlated with changes in exercise performance is consistent with the findings of earlier studies. We previously reported that moderate-intensity exercise can increase the abundance of *Prevotella*, a bacterium involved in carbohydrate metabolism and associated with enhanced glycogen synthesis capacity, in the mouse intestinal tract (Oliveira et al., 2022). In the current study, transplanting a microbiota suspension from Group E to Group MT significantly increased the *Firmicutes* abundance and the F/B ratio, which is associated with energy absorption efficiency (Ley et al., 2006) and could explain the improvement of exercise capacity in Group MT. In addition, PICRUSt analysis showed that pathways such as bacterial chemotaxis and flagellar assembly, which are related to bacterial colonization ability and intestinal mucosal barrier function (Laganenka et al., 2023; Kostic et al., 2015), were increased in MT-FMT. Furthermore, the flavone and flavonol biosynthesis pathways were upregulated, and flavonoids have antioxidant and anti-inflammatory effects, which may be indirectly involved in improved exercise capacity (Ji et al., 2024; Douae et al., 2025). These results suggest that exercise may provide the host with more energy and antioxidant substances by regulating the metabolic pathways of intestinal microbiota, thereby improving exercise capacity.

A key finding from our study is that transplanting the intestinal microbiota of mice subjected to exercise training can significantly change the intestinal microbiota structure of recipient mice and improve their exhaustive exercise capacity; however, the effect is not as strong as that seen with a direct exercise intervention. This finding suggests that the intestinal microbiota is an important mediator in exercise capacity improvement, but is not the only factor. This conclusion is consistent with the findings of Scheiman et al., who demonstrated that transplanting *Veillonella* can improve exercise endurance of mice, but that the effect is still weaker than that of long-term exercise training (Scheiman et al., 2019). It is possible that fecal microbiota transplantation did not fully replicate the characteristics of the Group E microbiota, resulting in insufficient abundance of some exercise-related dominant bacteria. Furthermore, the direct effects of exercise, such as muscle fiber type conversion and enhanced mitochondrial function (Zhang et al., 2025; Holani et al., 2024), cannot be transmitted through the microbiota. Moreover, the efficiency of transplanted microbiota colonization of the recipient intestine is limited and may be influenced by the recipient’s intestinal environment, such as pH and bile acid levels (Wexler et al., 2021). There are a few possible mechanisms for the improvement in exercise capacity that we observed in Group MT. Metabolites produced by the microbiota may play a role; for example, *Clostridium* and *Ruminococcus* can ferment dietary fiber to produce SCFAs such as propionate, which improves exercise endurance by inhibiting lactic acid accumulation and regulating energy metabolism (Scheiman et al., 2019), and butyrate, which enhances intestinal barrier function, reduces endotoxin entry into the blood, and alleviates inflammation (Huang et al., 2021; Mishiro et al., 2013). The intestinal microbiota may also interact with host signaling pathways to regulate metabolism in the liver and muscles, for example, through the vagus nerve or immune factors, thereby promoting glycogen synthesis and the expression of antioxidant enzymes (Sittipo et al., 2022). Another possible explanation is that exercise alters the abundance of certain genera in the intestinal microbiota, leading to changes in their metabolite levels that affect host signaling pathways. For example, the increased abundance of *Bifidobacterium* in Group MT may reduce intestinal lipopolysaccharide levels, thereby reducing inflammation and alleviating muscle oxidative damage. The increased abundance of *Ruminococcus* in Group MT could lead to increased fermentation of dietary fiber and, consequently, increased propionate production, which promotes GLUT4 expression in skeletal muscle by activating G protein-coupled receptor 41 (GPR41)/G protein-coupled receptor 43 (GPR43), resulting in accelerated glucose uptake (Han et al., 2014; Hong et al., 2005; Tokach et al., 2015). This finding is consistent with the increased muscle glycogen levels and exhaustive exercise capacity observed in Group MT in this study. Future studies should explore the specific mechanism(s) by which the microbiota contributes to increased exercise capacity.

### 4.1 This study has some limitations

First, microbiome analysis was performed using an OTU-based approach, whereas the current mainstream ASV methods (e.g., DADA2) can achieve higher-resolution sequence resolution (e.g., distinguishing sequence variants with single-base differences). We did not directly detect intestinal microbiota metabolites; thus, we could not clarify the specific molecular mechanism by which the microbiota affects exercise capacity. In addition, the long-term effect of exercise on the intestinal microbiota was not analyzed. Furthermore, only male mice were used; thus, the effect of sex differences on the exercise–microbiota interaction was not investigated. Future studies can use the ASV method to further explore the fine changes of the microbiota; employ metabolomics technology to detect metabolites such as SCFAs and lactic acid in the feces and serum to clarify the association between microbiota metabolism and exercise capacity; verify the regulatory effect of specific functional bacteria (such as *Veillonella* and *Clostridium*) on exercise capacity through antibiotic treatment and microbiota reconstitution experiments; explore the synergistic effect of both exercise and microbiota alteration on exercise capacity; and include female mice to determine the effect of sex differences on the exercise–microbiota interaction, thereby providing a more comprehensive basis for clinical application.

In summary, 14 weeks of moderate-intensity long-term endurance exercise significantly increased hepatic and muscle glycogen reserves, reduced hepatic lipid peroxidation levels, enhanced liver and gastrocnemius antioxidant capacity, and improved exercise-related metabolic functions in C57BL/6 mice. Long-term endurance exercise also significantly altered intestinal microbiota structure and function in mice, including increased *Firmicutes* abundance and αdiversity, enrichment in beneficial genera such as *Bifidobacterium*, *Ruminococcus*, and *Clostridium*, reduced abundance of harmful genera such as *Parabacteroides* and *Sutterella*, and enhanced activity of metabolic pathways such as the SCFA synthesis and flavonoid biosynthesis pathways. This study quantified the associations of S*utterella* and *Dorea* with exercise capacity in mice, providing new targets for subsequent targeted bacterial interventions. Previous studies have explored the associations between the intestinal microbiota and exercise-related phenotypes (Dowden et al., 2020; Yun et al., 2024), and the present study further focuses on the quantitative relationship between specific genera and exercise capacity, supplementing the evidence chain of the “exercise-microbiota-exercise capacity” regulatory axis. These exercise-induced changes in the intestinal microbiota could be partially transferred via FMT, with the effect weaker than that of a direct exercise intervention, indicating that the gut microbiota is a partial mediator of exercise capacity improvement (alongside skeletal muscle adaptation and mitochondrial function). Thus, our study confirms that the intestinal microbiota is an important mediator of exercise capacity and provides new avenues for future research on exercise training and health promotion.

### 4.2 Perspective

Our findings are highly relevant to the effects of physical activity on health and disease in sports medicine, as they provide insight into how the body and its resident microbiota work together to adapt to increasing performance demands. The results from our study are consistent with an earlier study showing that moderate-intensity physical activity increased the abundance of short-chain fatty acid-producing microbes in middle-aged women compared with sedentary behavior (Pérez-Prieto et al., 2024). However, another study showed that competing in a multi-day soccer tournament did not change the gut microbiota composition of female athletes (Oliveira et al., 2022), suggesting a complex relationship between exercise intensity and microbiota changes. Indeed, one study showed that excessive exercise in mice decreased gut microbial diversity (Yuan et al., 2018). Thus, our study provides further evidence that the gut microbiota is primarily altered by moderate exercise intensity and that this effect is sustainable and transferable.

The authors declare no conflicts of interest. The results of the study are presented clearly, honestly, and without fabrication, falsification, or inappropriate data manipulation. The results of the present study do not constitute endorsement by the American College of Sports Medicine.

## Data Availability

All raw 16S rRNA gene sequencing data generated in this study have been publicly deposited in the Sequence Read Archive (SRA) database of the National Center for Biotechnology Information (NCBI). The dataset of the control group and long-term endurance exercise group is available under the accession number PRJNA734109 (https://www.ncbi.nlm.nih.gov/bioproject/PRJNA734109), in which sample NC5 corresponds to the control group and sample NE5 corresponds to the long-term endurance exercise group. The sequencing data of fecal microbiota transplantation-related samples (PBS transplantation group and FMT group at pre-intestinal clearance, post-intestinal clearance, and post-transplantation stages) are available under the accession number PRJNA1469782 (https://www.ncbi.nlm.nih.gov/bioproject/PRJNA1469782), including PT1/PT2/PT3 for the PBS transplantation group and MT1/MT2/MT3 for the FMT group at corresponding time points.
