# Supplementary material for: Long-term endurance exercise influences exercise capacity in mice through modulation of the intestinal microbiota by fecal microbiota transplantation
Source: Front Microbiol. 2026 Jun 29;17:1830033. doi: 10.3389/fmicb.2026.1830033 (PMC13357625; doi:10.3389/fmicb.2026.1830033)
Supplement: Supplementary file 1 [file Supplementary_file_1.DOCX]

Table1 Summary of sample sequencing results

|  | **Pre-Intestinal Clearance (PT-Pre/MT-Pre)** | | | **Post-Intestinal Clearance (PT-Post/MT-Post)** | | | **Post-FMT**  **(PT-FMT/MT-FMT)** | | |
| --- | --- | --- | --- | --- | --- | --- | --- | --- | --- |
|  | **tags** | **clean_tags** | **OTU** | **tags** | **clean_tags** | **OTU** | **tags** | **clean_tags** | **OTU** |
| sum | 3,001,632 | 2,907,558 | 105,813 | 2,925,699 | 2,866,973 | 2673 | 2,899,676 | 2,798,784 | 83,224 |
| min | 108,467 | 102,154 | 3360 | 119,293 | 115,299 | 53 | 100,459 | 95,251 | 2200 |
| max | 139,964 | 138,321 | 5971 | 139,996 | 139,090 | 281 | 139,935 | 137,860 | 5441 |
| mean | 136,438 | 132,162 | 4810 | 132,986 | 130,317 | 122 | 131,803 | 127,217 | 3783 |
| SD | 9101 | 9773 | 801 | 6174 | 6365 | 57 | 12,132 | 12,540 | 898 |

Note: sum, total number of sequences; min, minimum value; max, maximum value; mean, average value; SD, standard deviation; tags, usable sequences; clean_tags, effective sequences; OTU, operational taxonomic unit.

Table 2 Pairwise comparisons of alpha diversity indices (Shannon, Simpson, and Chao1) among six groups

| **Shannon** | | | | | |
| --- | --- | --- | --- | --- | --- |
|  | PT-Pre | PT-Post | PT-FMT | MT-Pre | MT-Post |
| PT-Post | ** | NA | NA | NA | NA |
| PT-FMT | ** | ** | NA | NA | NA |
| MT-Pre | ns | ** | ** | NA | NA |
| MT-Post | ** | ** | ** | ** | NA |
| MT-FMT | ns | ** | ** | ns | ** |
| **Simpson** | | | | | |
|  | PT-Pre | PT-Post | PT-FMT | MT-Pre | MT-Post |
| PT-Post | ** | NA | NA | NA | NA |
| PT-FMT | ** | ** | NA | NA | NA |
| MT-Pre | * | ** | ** | NA | NA |
| MT-Post | ** | ** | ** | ** | NA |
| MT-FMT | ns | ** | ** | ns | ** |
| **CHAO1** | | | | | |
|  | PT-Pre | PT-Post | PT-FMT | MT-Pre | MT-Post |
| PT-Post | ** | NA | NA | NA | NA |
| PT-FMT | ** | ** | NA | NA | NA |
| MT-Pre | ns | ** | ** | NA | NA |
| MT-Post | ** | ns | ** | ** | NA |
| MT-FMT | * | ** | ns | ns | ** |

Note: The table presents FDR-corrected P values for pairwise comparisons of alpha diversity indices (Shannon, Simpson, Chao1) across the six groups (PT-Pre, PT-Post, PT-FMT, MT-Pre, MT-Post, MT-FMT) using the Wilcoxon rank-sum test. NA: Not applicable (no comparison performed). Significance levels: **P < 0.01, *P < 0.05, and ns (not significant, P ≥ 0.05).


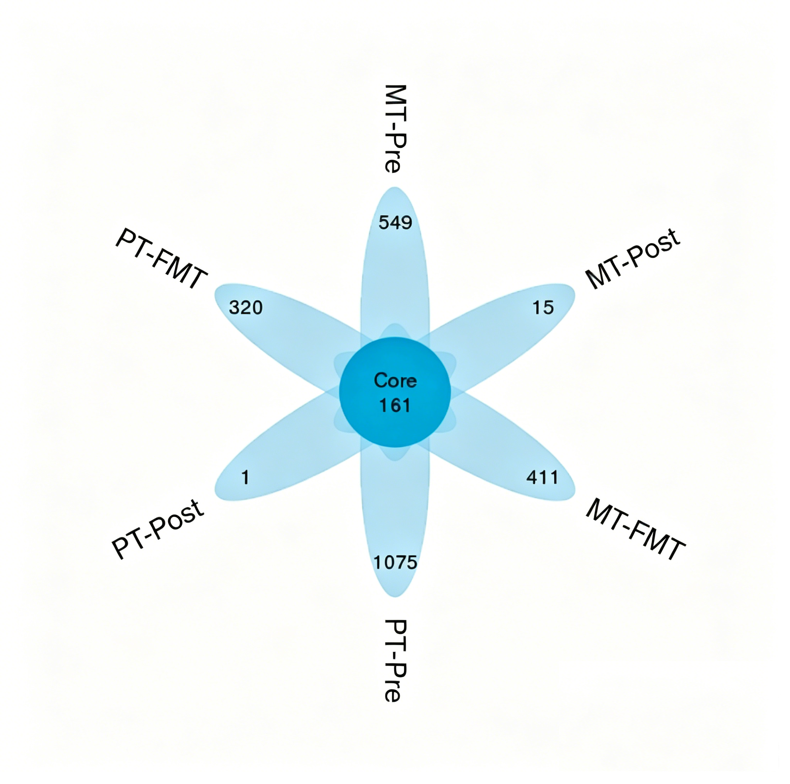


Figure 1 Venn diagrams of the PT and MT groups at different stages. Each blue area represents a group, and the overlapping areas between the blue areas represent shared OTUs between groups.


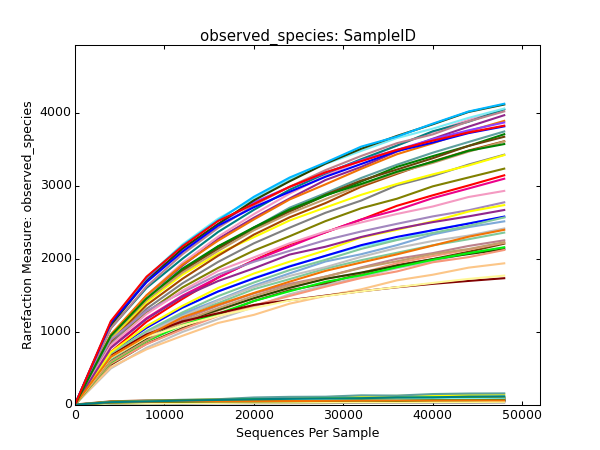

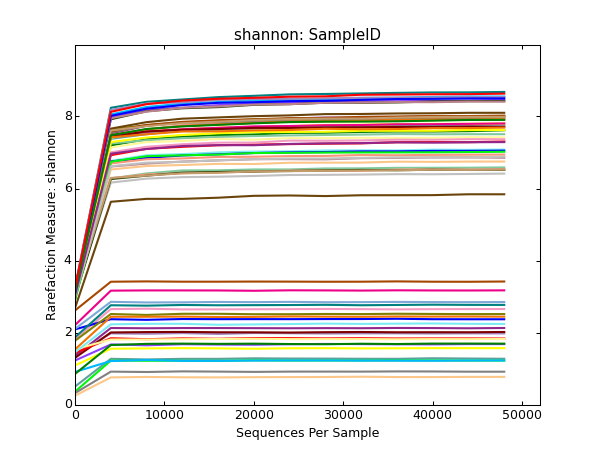


Figure 2 Rarefaction curves and Shannon-Winner curves. (A) Rarefaction curves of observed species. The observed_species index represents the actual number of observed OTUs. The x-axis is the number of randomly selected sequences per sample, and the y-axis is the number of OTUs at the corresponding depth. (B) Shannon-Winner curves. The x-axis represents the number of randomly selected sequences, and the y-axis represents the Shannon index reflecting species diversity. Each color in the figure represents a single sample.
